# The Differences of Natural Endowment

**Published:** 1833-04

**Authors:** 


NATURAL ENDOWMENT.

### *The Differences of Natural Endowment.* By J. H., Esq. Surgeon.

IT is a melancholy fact in the history of science, that truth has generally been received by mankind with reluctance and aversion; and, indeed, so supported by evidence is this observation, that the opposition engendered has almost invariably been in proportion to the importance and merits of the discovery. The attention of many has been attracted to the science of Phrenology by the virulence of its opponents, who, in evincing a stronger disposition to malign its supporters than to offer the result of their own investigations on the subject, have afforded a special though negative argument in its favour. By far the greater number of those who have thought it expedient to revile phrenology, have scarcely condescended to acquaint themselves with the rudiments of the science, or they would not have charged it with doctrines which its founders have never advanced.

Nevertheless, there appear to be some who have rather mistaken than wilfully misconstrued the interpetration of its doctrines, and there are those who believe that it inculcates the dogma of utter inability in man to control the propensities and feelings of his nature. Now this is a tenet which the doctrines of phrenology more decidedly tend to controvert, than any other scheme of moral philosophy that metaphysicians have ventured to uphold from the commencement of the Christian era to the present day; inasmuch as it is the first and only system that has ever established education on the rational and substantial basis of adapting the means to the end; in other words, of regulating instruction, habits, and employment, by and in accordance with the moral and intellectual endowments of individuals; that is, of cultivating the mind after such a manner as will insure the greatest proportion of knowledge, and promote that mental discipline which alone can enable us to control and direct these natural feelings and propensities. The great advantage derivable from phrenology consists in the increased power it hath given us, through the medium of education, of encouraging the action of the moral faculties to the better guidance and subordination of the lower. It is phrenology that raises education to the dignity of a science, which, if we cast but a glance on the numberless and various opinions of celebrated men upon the subject, appears to have hitherto been founded on remarkably vague and indefinite principles.

Having premised these few observations, we turn our attention to the consideration of the more immediate subject of this article, namely, the differences of natural endowment. We have heard it maintained, "that all men are born with the same feelings, and that education and external circumstances alone create the difference in the power of controling those feelings within the bounds of morality;" again, "that all men are equally capable by nature of attaining the same degree of moral excellence, if they have similar advantages from education and external circumstances;" also, "that no man possesses by nature a nobler cast of moral feelings than another." All these positions are in effect the same, and an argument against one is an argument against all. As phrenology is a science entirely founded on observation and induction, it is to these that we shall resort in the consideration of the above opinions. It is an acknowledged fact that peculiarities of temper in children may be observed before external circumstances could possibly have exercised any imaginable influence upon them. For instance, let any one remark the difference of cry and manner in various children, even within a few months after birth, upon the first occasion of their being detained from the breast by any accidental circumstance. Will the expression of all be the same? no; some will moan patiently and piteously, and others will work themselves into a state of irritation beyond all power of pacification. But it may be said, that this is owing to the constitutional disposition of some to feel hunger more acutely than others; granted that it may occasionally; yet upon the testimony of many parents who have possessed these irritable children and watched their dispositions in after life, we are warranted in concluding that it arises from temper of mind far more frequently than constitution of body. "That all men are born with the same feelings" is true in regard to number, but not with respect to degree, which may be best ascertained by observing the differences of character that exist among the inmates of such institutions as the Hôpital des Enfans Trouvés in Paris, where all are as nearly as possible subjected to the same kind of habits and education from the period of infancy. Here you will find some of a proud, wilful, and obstinate disposition; others of a meek, patient, and submissive nature: some of a cunning, sly, crafty, and deceitful character; others of a frank, free, open, and generous spirit: some of strong animal courage, though of delicate frame; and others as timid as they are robust: here you may mark one firm and inflexible of purpose; there you may see another fickle, uncertain, and inconstant of conduct: one will regard the sufferings of animals with satisfaction, and torment them for his gratification, while another, of a kind and benevolent heart, will shrink with horror and aversion from the sight: in some the animal passions will develop themselves early in life, and acquire a strength in maturer age that will cause the individual frequently to gratify or control them: while in others they are developed at a much later period, and in so moderate a degree as to seldom excite the attention or care of their possessor. Finally, we appeal to common observation and experience in support of the opinion now advanced, that there are some who from earliest youth are possessed of so strong a sense of morality that they shrink from vice as naturally as from a venomous reptile, and require an extraordinary inducement, a prolonged and seductive temptation, to bend them from their innate leaning: on the contrary, that there are others who from childhood upwards exhibit such strong propensities to do evil, that the strictest possible discipline, combined with the best education, can scarcely prevent their errors: and thirdly, that there are all the gradations of disposition between these two extremes. Now, men of these different casts of character could not be rendered *equally capable* of attaining moral excellence by means of the same education and external circumstances. To say that they would, is to affirm that the *same thing* will have the *same effect* upon two *dissimilar things,* which is an absurdity.

It is not uncommon in large families, where education and external circumstances have varied but little with respect to the children, to find one that is an absolute blot upon the credit of the others; nay, this one perhaps, on account of the perverseness of his nature, may himself have been the subject of a stricter and better education than the rest. These are no fanciful statements, such instances are frequently to be observed. Observation, therefore, has induced phrenologists to believe that men differ from each other in the endowment of *moral feeling,* as well as in *intellectual capacity.* That they differ in the latter is acknowledged by all; that they do so in the former is no less evident, though less frequently admitted. Granting, however, for the sake of argument, that men did not differ in moral feeling, but in intellectual capacity only, will not the strong and vigorous reasoning of a powerful mind create such motives for, and perceive that advantage in a system of well-regulated conduct, which the weak and feeble intellect is ever blind to? Can the intelligent being and the dull one be equally advantaged in a moral sense by the influence of education? We acknowledge that, if each be subjected to the power of ill instruction and evil example, the intellectual being, inasmuch as he is capable of a wider sphere of action, may probably become a more vicious member of society than his dull brother. Nevertheless, should both enjoy equal chances of deriving advantage from moral and intellectual discipline, we think it will be found (with submission to more experienced judgment,) that the individual of the greatest capacity will become the better man, in proportion to his superior power of receiving instruction and knowledge. Doubtless he will be able to reflect more soundly upon vice and its consequences than a person of contracted understanding; and the simple capability of regarding vice in its true colours is alone sufficient to engender a powerful motive for avoiding it; for the mind that possesses the power of looking upon vice as it is, knows it to be adverse to the happiness and interest of man not only hereafter but here. Who practices morality the most, seeks his own interest the best. We conclude, therefore, that the intellectual powers alone, if well cultivated, will offer motives to a man for the regulation of his passions.

Another point, too, is involved in the positions under consideration, namely, that of giving the same education indiscriminately to all. This would be to act in contradiction to the almost self-evident principle, that the same education, moral and intellectual, is by no means equally well adapted to all dispositions. The slow cannot learn with the quick; the same measures should not be used with the proud boy as the meek, or with the benevolent as the cruel, or with the diffident as the forward, or with the artful and cunning as with the open and sincere, or with the conscientious mind as with one less scrupulous. The same education and habits being, therefore, unequally fitted for the improvement of all, ought not to be employed for that purpose. All are not to be induced by the same motive to pursue the same end. On this account the positions are a second time inconsistent with reason.

Though all soils may be so cultivated and improved as to produce something, the product of each will not be of equal quality and value. Neither do phrenologists believe, for the reasons above stated, that education and example, or any means whatever, would elevate all men's minds to an equal standard of moral excellence. We are perfectly convinced of the powerful influence of education upon man, and fully aware of the extreme importance of establishing that science upon rational and well-considered principles. Well do we also know that nothing will so directly tend to increase and encourage virtue in all as good instruction and example; man has indeed no other means of improvement. But to affirm that the same education and external circumstances will make all men equally capable of practising morality, is almost as absurd as to say that the same training will make all horses equally fleet. When men are all formed by nature with the same dispositions, same constitutions, same brains, and same forms, the same education will have upon them the same effect, but not till then.

And now do we go to consider how far these views of the rational laws of man affect what is called free agency. It is not to be understood that the varying natural capability in men to attain moral excellence, implies that some are altogether unable to suppress their vicious inclinations. This, we repeat, is by no means a necessary consequence, though the opponents of phrenology have found it convenient to urge it against us. On the contrary, the position we assume as a general rule is this, that well-conducted education will enable all men to restrain their passions and propensities within the bounds of morality, but not with *equal facility*, inasmuch as some possess stronger propensities and passions than others, and this in combination with a more limited proportion of the moral sentiments. To what extent then do these views affect free agency? Why, abstractedly considered, they leave it as they found it, showing only that, though it exist in all, it *doth not exist to the same degree in* all; and this is our creed, a creed which our opponents have proved exceedingly unwilling to refrain from perverting. However, as man is more prone to evil than good, and education in general but defectively conducted, it is not surprising that selfishness and immorality predominate in the world.

But it is contrary to the justice of Providence and to Christianity, cry our opponents, that some men should possess a greater natural capability of practising morality than others. With respect to the justice of the matter, we have only to remark, that observation and induction, the only means in our power of obtaining any species of knowledge whatever, supplies every evidence in proof of the fact which such a fact is capable of bearing. On the principle, therefore, that a natural phenomenon can never warrant us in arraigning the justice of the Almighty, we dismiss the implication. As regards the charge of the fact being in contradiction with Christianity, we are not aware that it has ever been attempted to be proved, though many have made the assertion. When our opponents think proper to state the grounds upon which they found this charge, it will be time enough to reply to it. Nevertheless, we give it as our opinion that Christianity, as well as nature proclaims original differences among men. It allows that some are more, others less, gifted; but it makes each answerable only for the gifts he has received: to whom much is given, from him shall much be required. St. Paul observes, "for I would that all men were even as I myself: but *every man hath his proper gift of God, one after this manner, and another after that.*" (1 Cor. vii. 7.)

Let the reader who thinks that this sentence is used in relation to intellect alone, refer to the chapter from which it is taken. Again, we are exhorted by the same, "to comfort the *feeble-minded*, support the *weak*, and be patient toward all." (1 Thessal. v. 14.) Thirdly, in the parable of the sower and his seed, "but that which fell on good ground are they, which in "an *honest and good heart*, having heard the word, keep it, and bring forth fruit with patience." (Luke, viii. 15.) Fourthly, "for there are some eunuchs which were *so born* from their mother's womb; and there are some eunuchs which were made eunuchs of men; and there be eunuchs which have made themselves eunuchs for the kingdom of heaven's sake." (Matthew, xix. 12.) This last quotation relates to the varying strength of an animal passion in man, the ill government of which is productive of no small proportion of the vice that exists in the world. We are well aware it will be said that Scripture may be quoted for any purpose, but with such futile remarks as these our opponents neither strengthen their own positions, nor weaken ours. Let them rather show how we have misinterpreted the Scriptures, and endeavour to prove the contrary from them. If they but fail in the latter, the positions we have maintained in this article will have a right to stand as proved on the authority of nature alone, for it by no means follows that all which is not especially expressed in revelation must be in contradiction with it.

Be it then specially understood that phrenologists inculcate no such doctrine as irresistibility of action; though they insist on the innateness of the faculties, and the differences of moral as well as intellectual endowment amongst mankind. We are inclined to believe, indeed, that they perceive more distinctly than any other sect of moral philosophers the truth of the following observations: "C'est l'abus de nos facultés qui nous rend malheureux et mechants. Nos chagrins, nos soucis, nos peines, nous viennent de nous. Le mal moral est incontestablement notre ouvrage, et le mal physique ne serait rien sans nos vices qui nous l'ont rendu sensible."

